# Phylogenic analysis of human bocavirus detected in children with acute respiratory infection in Yaounde, Cameroon

**DOI:** 10.1186/s13104-017-2620-y

**Published:** 2017-07-17

**Authors:** Sebastien Kenmoe, Marie-Astrid Vernet, Mohamadou Njankouo-Ripa, Véronique Beng Penlap, Astrid Vabret, Richard Njouom

**Affiliations:** 1 Virology Unit, Centre Pasteur of Cameroon, Yaounde, BP 1274 Yaounde, Cameroon; 2 Biochemistry Department, Université of Yaounde 1, Yaounde, Cameroon; 3 Virology Service, Pôle de Biologie, CHU de Caen, Caen, France

**Keywords:** Human bocavirus, Children, Acute respiratory infection, Cameroon, Africa

## Abstract

**Objective:**

Human Bocavirus (HBoV) was first identified in 2005 and has been shown to be a common cause of respiratory infections and gastroenteritis in children. In a recent study, we found that 10.7% of children with acute respiratory infections (ARI) were infected by HBoV. Genetic characterization of this virus remains unknown in Central Africa, particularly in Cameroon Leeding us to evaluate the molecular characteristics of HBoV strains in Cameroonian children with ARI.

**Results:**

Phylogenetic analysis of partial HBoV VP1/2 sequences showed a low level of nucleotide variation and the circulation of HBoV genotype 1 (HBoV-1) only. Three clades were obtained, two clustering with each of the reference strains ST1 and ST2, and a third group consisting of only Cameroon strains. By comparing with the Swedish reference sequences, ST1 and ST2, Cameroon sequences showed nucleotide and amino acid similarities of respectively 97.36–100% and 98.35–100%. These results could help improve strategies for monitoring and control of respiratory infections in Cameroon.

## Introduction

During the past two decades, new molecular techniques have contributed to the discovery of many viruses. This was the case in September 2005 with the discovery of the Human Bocavirus (HBoV) by a Swedish research team [1]. HBoV was subsequently named HBoV-1. Since, three other species (HBoV-2, 3, and 4) have been described from stool samples in Pakistan, Australia, and Nigeria respectively [2–4]. Based on phylogenetic analysis of HBoV genomic sequences, it was assigned to the *Parvoviridae* family, *Parvovirinae* sub-family, and Bocavirus genus. Indeed, the sequences identified were closely related to two other bocaviruses: the Bovine Parvovirus and the canine Minute Virus. Although this classification was based on genetic characterization, microscopy studies have observed viral particles having the characteristics of this family of viruses; small DNA viruses with unenveloped icosahedral capsid and a diameter ranging from 18 to 26 nm. The HBoV genome is a 5.2 kb linear negative sense single-stranded DNA. During the 8th ICTV report it was decided that the different species of the genus Bocavirus should show a similarity in the NS1 gene of at least 95%. Based on the analysis of a large number of VP1 gene sequences and calculations of the genetic distance of complete genomes of different species of HBoV, Kapoor and collaborators recently proposed a new classification mode for HBoV suggesting that HBoV strains with more than 8 and 10% difference respectively in protein and nucleotide sequences of the VP1 complete gene should be considered as different species. Those with more than respectively 1.5 and 5% difference should be considered as different genotypes [3]. This proposal is consistent with the current nomenclature of the four HBoV species. A decade after the identification of HBoV-1 in Sweden, it has been detected at frequencies ranging from 2 to 19% [5] in several countries across Europe [6–9], Asia [10–13], America [14–17], Africa [18–21], Middle East [22–24], and Australia [4, 25], revealing an overall distribution of this virus. These studies indicate that HBoV-1 is primarily a respiratory pathogen. HBoVs are detected not only in the respiratory tract (HBoV-1 and 2), but also in the gastrointestinal tract (HBoV-1 to 4) where they are associated with gastroenteritis [5]. HBoV are highly conserved viruses and sequences obtained in various regions of the world by several groups highlight a high homology of genetic sequences [7, 9, 11, 18, 22].

In a recent study, we have found that 10.7% of children with acute respiratory infections (ARI) were infected by a HBoV [21]. However, most molecular epidemiology studies of HBoVs have been performed in developed countries [5]. Genetic characterization of this virus remains unknown in most African countries, particularly in Cameroon. This study is, to our knowledge, the first analysis of the genetic characteristics of HBoVs obtained from Cameroonian children with acute respiratory infections (ARI).

## Methods

### Study population and samples

The study population was hospitalized and outpatient children with ARI consulting at the pediatric service of ‘Centre Hospitalier d’Essos’ in Yaounde, Cameroon. Demographic and clinical characteristics of these children have been previously described [21]. For this study nasopharyngeal swab samples were used.

### Laboratory analysis

DNA was extracted using QIAamp DNA Mini kit (Qiagen, Hiden, Germany, Cat No. 51306) from nasopharyngeal swab specimens following the manufacturer’s instructions. A final elution volume of 200 µL of DNA was stored a −80 °C prior to testing by a commercially available duplex real time PCR for the detection of human Adenovirus and HBoV (Respiratory Multi Well System r-gene™, BioMerieux, Lyon, France, Cat No. 1092480) following the manufacturer’s instructions. HBoV positive samples were then selected and a fragment of the VP1/2 capsid gene was amplified by semi-nested PCR as described elsewhere [18]. Briefly, 10 µL of the DNA was added to 50 µL of PCR mixture containing of 24.5 µL of water, 1.5 µL of MgCl_2_ at 50 mM, 1 µL dNTPs at 10 mM, 2,5 µL of VP-A sense primer (5′-GCACTTCTGTATCAGATGCCTT-3′) at 10 µM, 2.5 µL of VP-B reverse primer (5′-CGTGGTATGTAGGCGTGTAG-3′) at 10 µM, 5 µL of 10× PCR buffer, 2.5 µL of the W1 solution at 1%, and 0.5 µL of the Taq DNA Polymerase (Invitrogen, USA, Cat No. 10342053). To improve the sensitivity, a second semi-nested reaction with 2 µL of the first PCR product was performed with the pair of primer VB-B and VP-C (5′-CTTAGAACTGGTGAGAGCACTG-3′) according to the previous protocol. The cycling parameters for the two PCR included an enzyme activation and initial denaturation at 94 °C (3 min); 40 cycles of denaturation at 94 °C (45 s), primer annealing at 50 °C (30 s) and extension at 72 °C (1 min 30 s); a final extension at 72 °C (10 min), and a hold at 4 °C. The amplified VP1/2 capsid of 980 bp product was observed after electrophoresis in a 1.5% agarose gel.

The VP1/2 capsid PCR products were purified and sequenced using Big Dye Terminator cycle sequencing (Applied Biosystems, Foster City, USA, Cat No. 4,337,454) and semi-nested PCR primers according manufacturer instructions. Electrophoresis and data collection were done on an Applied Biosystems AB3100 genetic analyzer. Sequences were first aligned and edited using Sequence Navigator1 software (Applied Biosystems, Foster City, CA). The consensus sequence was generated by removing low quality base peaks at the end of the chromatogram and correcting base pair mismatches.

### Phylogenetic analysis

Genotypes were determined by phylogenetic analysis comparing the consensus sequence of each sample to reference VP1/2 capsid sequences including the Swedish reference sequences ST1 and ST2, and reference sequences of HBoV-2 (NC_012042), HBoV-3 (NC_012564), HBoV-4 (NC_012729), BPV (NC_001540.1), and MVC (NC_004442.1).

Phylogenetic analyses were performed using MEGA software version 6 [26]. Briefly, sequences in FASTA format were aligned using Clustal W algorithm. Genetic distances were measured with the Kimura-2 parameter model. A phylogenetic tree was constructed with the neighbor-joining algorithm and robustness of the tree was evaluated with 1000 bootstrap resamplings.

The MEGA software version 6 was used to generate predicted amino acid sequences from consensus nucleotide sequences by translation. The comparison of the sites and proportion of similarities at amino acid and nucleotide levels was made between consensus amino acid and nucleotide sequences of each Cameroonian HBoV sequences and ST1 and ST2 reference sequences.

All consensus nucleotide sequences obtained in this study were submitted to GenBank Database under accession numbers KX121135 to KX121163.

## Results

### Genotyping of HBoV isolates

**Fig. 1:**
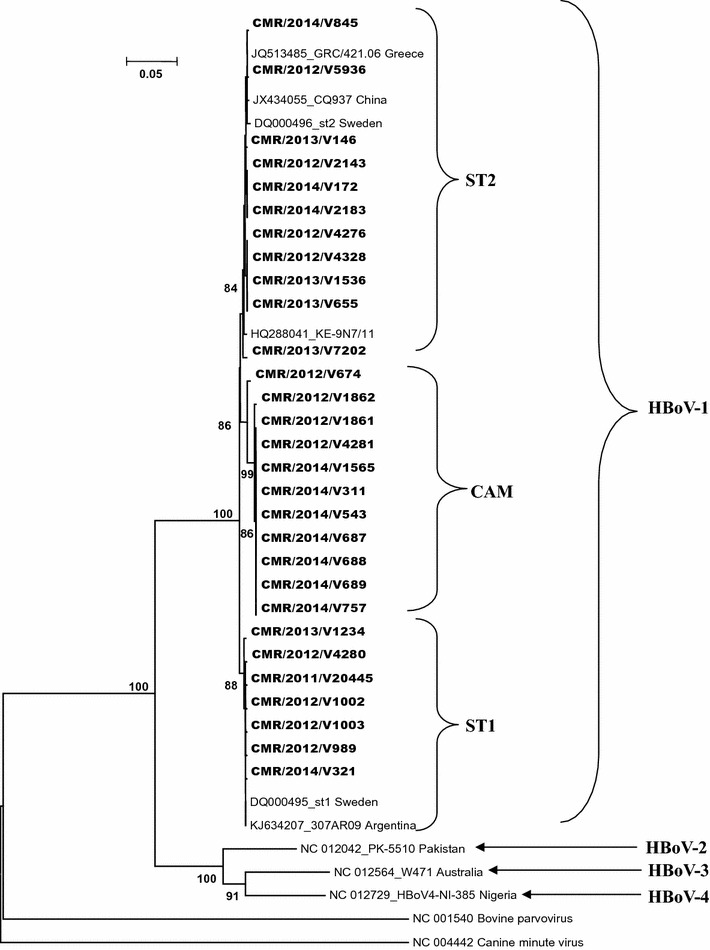
Neighbor-joining phylogenetic tree of the 29 human bocavirus strains from Cameroon, partial VP1/2 gene (839 bp). Sequences from this study are in *bold*. The number next to the node are bootstrap values. Only bootstrap values higher than 80% are presented. ST1 are sequences clustering with the reference sequence ST1. ST2 are sequences clustering with the reference sequence ST2. CAM are the Cameroon cluster group

Among the 80 HBoV positive samples, DNA in the VP1/2 capsid region could be amplified, sequenced and analysed phylogenetically in 29 samples (36.2%). The estimated phylogeny of these sequences and previously published VP1/2 capsid sequences including those of the four species of HBoV is shown in Fig. 1. All of the 29 sequences clustered with HBoV-1 reference sequences. Three clades were obtained, two clustering with each of the reference strains ST1 and ST2, and a third group consisting of only Cameroon strains (CAM).

By comparing with the Swedish reference sequences ST1 and ST2, Cameroon sequences showed nucleotide and amino acid similarities of respectively 97.36–100% and 98.35–100%.

### Nucleotide and aminoacid polymorphisms

Based on the VP1/2 capsid gene of the Swedish reference strain (accession number NC_007455), 28 of 717 nucleotides analyzed (position 1141–1857 of VP1/2 capsid gene) were found to be variable, with 21 (75%) transitions and 7 (25%) transversions. Ten nucleotide substitutions (G1243A, C1429T, G1470A, A1539G, C1542T, A1551G, T1619A, A1636C, T1800C, and A1821G) were specific to the Cameroonian clade: CAM (data not shown and available upon request). The description of the amino acid sequences deduced from HBoV-1 strains of this study was made with reference to the VP1/2 capsid domain of HBoV reference (accession number NC_007455), which extended over a length of 239 amino acids (position 381–619). The HBoV strains analyzed in the study had the amino acid substitutions: G415S, N474S, F540Y, N546H, and S590T (data not shown and available upon request). Three of these amino-acid substitutions (G415S, F540Y, and N546H) were specific to the CAM clade.

## Discussion

This study reports for the first time molecular characteristics of HBoV in children suffering from acute respiratory infections in Cameroon. Only 29 (36.2%) tested samples were amplified by genotyping PCR on the VP1/2 capsid gene during this study. This low amplification rate can be explained by the high sensitivity of the detection technique compared to the genotyping technique, and also by the low viral load in unamplified samples (mean cycle threshold unamplified samples = 35.6 ± 1.8 vs. 28.1 ± 6.3 for the positive, p < 0.001). Several studies have shown that HBoV-1 is a respiratory pathogen [5]. As expected, phylogenetic analysis of partial VP1/2 capsid gene sequences obtained in this study also revealed the presence of the HBoV-1 species. These viruses circulating among children with acute respiratory infections in Cameroon were divided into two genetic clades closely linked to ST1 and ST2 and a third clade made only of Cameroonian strains. The sequences revealed an extremely high homology with the original sequences (ST1 and ST2) identified in Sweden in 2005. These results are consistent with findings of other authors from around the world [6, 7, 23]. However, unlike these results and others [6, 7, 15–17, 23], over 10 nucleotide substitutions leading to three amino acid mutations were specific to the CAM clade, suggesting that HBoV undergo higher selective pressures in Yaounde, Cameroon than in other regions.

Our study included limited number of patients from a single health care facility and is therefore not representative of our country. However, our sequences are also similar to those circulating in other countries, highlighting the low genetic variability of HBoV. In summary, our data indicates that HBoV-1 strains collected from 2011 to 2014 in Yaounde are divided in three groups. Two groups clustered with previously reported ST1 and ST2 genotypes, and the third group is an original Cameroonian genotype. The partial HBoV-1 VP1/2 gene sequences in Cameroon have relatively high genetic variations compared to others studies.

## Limitations

Our study included limited number of patients from a single health care facility and is therefore not representative of our country.
